# Distributed large-scale graph processing on FPGAs

**DOI:** 10.1186/s40537-023-00756-x

**Published:** 2023-06-04

**Authors:** Amin Sahebi, Marco Barbone, Marco Procaccini, Wayne Luk, Georgi Gaydadjiev, Roberto Giorgi

**Affiliations:** 1 Department of Information Engineering and Mathematics, University of Siena, Siena, Italy; 2 Department of Information Engineering, University of Florence, Florence, Italy; 3 Department of Computing, Imperial College London, London, UK; 4 Department of Quantum and Computer Engineering, Delft University of Technology, Delft, Netherlands; 5 Consorzio Interuniversitario Nazionale per l’Informatica, Rome, Italy

**Keywords:** Graph processing, Distributed computing, Grid partitioning, FPGA, Accelerators

## Abstract

Processing large-scale graphs is challenging due to the nature of the computation that causes irregular memory access patterns. Managing such irregular accesses may cause significant performance degradation on both CPUs and GPUs. Thus, recent research trends propose graph processing acceleration with Field-Programmable Gate Arrays (FPGA). FPGAs are programmable hardware devices that can be fully customised to perform specific tasks in a highly parallel and efficient manner. However, FPGAs have a limited amount of on-chip memory that cannot fit the entire graph. Due to the limited device memory size, data needs to be repeatedly transferred to and from the FPGA on-chip memory, which makes data transfer time dominate over the computation time. A possible way to overcome the FPGA accelerators’ resource limitation is to engage a multi-FPGA distributed architecture and use an efficient partitioning scheme. Such a scheme aims to increase data locality and minimise communication between different partitions. This work proposes an FPGA processing engine that overlaps, hides and customises all data transfers so that the FPGA accelerator is fully utilised. This engine is integrated into a framework for using FPGA clusters and is able to use an offline partitioning method to facilitate the distribution of large-scale graphs. The proposed framework uses Hadoop at a higher level to map a graph to the underlying hardware platform. The higher layer of computation is responsible for gathering the blocks of data that have been pre-processed and stored on the host’s file system and distribute to a lower layer of computation made of FPGAs. We show how graph partitioning combined with an FPGA architecture will lead to high performance, even when the graph has Millions of vertices and Billions of edges. In the case of the PageRank algorithm, widely used for ranking the importance of nodes in a graph, compared to state-of-the-art CPU and GPU solutions, our implementation is the fastest, achieving a speedup of 13 compared to 8 and 3 respectively. Moreover, in the case of the large-scale graphs, the GPU solution fails due to memory limitations while the CPU solution achieves a speedup of 12 compared to the 26x achieved by our FPGA solution. Other state-of-the-art FPGA solutions are 28 times slower than our proposed solution. When the size of a graph limits the performance of a single FPGA device, our performance model shows that using multi-FPGAs in a distributed system can further improve the performance by about 12x. This highlights our implementation efficiency for large datasets not fitting in the on-chip memory of a hardware device.

## Introduction

Graphs are growing in popularity because they are a powerful tool for uncovering patterns, connections, and insights within data, which can be used to support a wide variety of applications, including fraud detection, social network, bio-informatics, and computer vision. Graphs are best at representing data with complex relationships and inter-dependencies often generated from non-Euclidean domains. Since these data are becoming more popular, graphs are growing in adoption, and recently, many studies on extending deep learning approaches for graphs have emerged. Especially graph neural networks (GNNs) [61]. Moreover, graph-based models are used heavily in the biological domain to predict the properties of new compounds, estimate their activity levels, predict their side effects [13], and generate candidate molecular structures [54]. However, due to their irregular nature, graphs are inherently hard to compute, becoming a challenging task [10, 23, 45, 46, 51, 66].

Graph computing is due to their irregular structure, which leads to a high amount of stochastic and irregular access to the memory [33]. This factor contributes to a lack of data locality, which leads to the inability to achieve high parallelism; multiple workers would only increase overhead instead of performance due to the contention on the memory controller. Recent advances in computer architectures offer different solutions for CPUs, GPUs, FPGAs, and other accelerators to overcome such computational challenges [20, 62, 64, 67, 72]. However, all these architectures present different trade-offs: GPUs, for example, are optimised for massively parallel workloads (e.g., Deep Neural Networks), while they are less efficient in applications containing extremely memory-sparse operations and data races issues [42, 65, 70]. Recently, reconfigurable architectures (e.g., Field Programmable Gate Array) are emerging as an attractive alternative to CPUs and GPUs for graph processing. Unlike traditional CPUs, which are designed to perform a wide range of tasks, FPGAs are highly customisable and can be tailored to perform specific functions. They can benefit from the dataflow execution model as it allows for highly parallel and efficient execution of tasks. In a dataflow execution model, tasks are broken down into small, independent units of work called “tasks” or “jobs”, which can be executed in parallel [27–30, 35]. This can be especially beneficial for graph processing tasks, as it allows for parallel execution of operations such as filtering, sorting, and memory access, allowing them to process large-scale graphs much more efficiently than CPUs or GPUs.

Additionally, FPGAs are highly efficient in handling memory-bound tasks, which is a common characteristic of graph processing algorithms [8, 16, 51, 70]. The increase in popularity of modern reconfigurable architectures made researchers focus more on mitigating the following problems:

- Graph size: in contrast to CPUs and GPUs, which use a cache hierarchy memory model, FPGAs can exploit various types of memory, including Lookup Tables (LUTs), Block RAM (BRAM), Ultra-RAM (URAM) and High-Bandwidth Memory (HBM). Unlike DRAM, these memory types can provide more performance thanks to their lower latency and higher bandwidth. Moreover, they can be fully customised and configured to suit the specific needs of the computation. For example, one of the most valuable features of BRAMs is their ability to provide high-throughput random access to memory [9, 26, 68]. The dual-port nature of the BRAM memories allows parallel, same-clock-cycle access to different locations [55]. However, this feature can also be a weakness of the system, as accessing the memory through the ports can cause a performance bottleneck. This is because the ports on BRAM are the only way to access the data stored within the memory. They are typically implemented using a finite number of physical connections. When multiple inputs or outputs are trying to access the memory simultaneously, they may have to wait for the ports to become available, which can delay and slow down the overall system performance. Another limitation of using FPGAs for large-graph processing is that the size of on-chip memory in a single FPGA is often not sufficient to store the entire size of the graph.
- Data locality: most of the graph structure is irregular, meaning that the degree of connectivity and the connection themselves may be highly different between nodes. This may considerably impact the performance as consequently accessed nodes might be stored far away in memory, hence causing cache misses that greatly degrade performance. As a result, in the vast majority of cases, data locality is a significant problem. Moreover, graphs are usually created based on a natural phenomenon, from Social Networks to Biological structures. Subsequently, the distribution of data in these graphs, instead of being uniform, follows the Power Law distribution, complicating the locality of data during the computation [18, 40];
- Irregular data access patterns: in unstructured graphs, the data access pattern is not predictable, meaning that each access to memory is done to a different position. This type of access is called irregular memory access. This makes it difficult to optimise memory access, as the location of data in memory is not known in advance, resulting in longer access times and leading to a decrease in performance [19, 69, 71]. Overall, in most graph processing applications, accessing irregular data is more time-consuming than the computation itself;
- Data conflicts: in graph applications, data conflicts are very common (e.g., reading/writing of the same vertex simultaneously). As a result, memory model policies, such as memory locks or atomic operations, are necessary to guarantee correctness, affecting the overall performance [66, 70] as they introduce overhead and limit parallelism.

The advent of big data resulted in an increase in model and dataset size and even graphs. Thus, such graphs, often called large-scale graphs, are not manageable by a single node anymore [44]. Hence, the advent of distributed large-scale graph computing. Many distributed frameworks can be used to analyse large-scale graphs, for example, Map-Reduce [5], Stratosphere [1], Hama [52], Giraph [50], Graphlab [41]. Although these frameworks are not capable of natively targeting accelerators, they offer good performance when used to analyse large-scale graphs [22].

This study targets the challenges of large-scale graph processing and proposes a new framework. This framework adapts the state-of-the-art partitioning scheme proposed in GridGraph [32] for FPGA use. The GridGraph library partitions the edges of a graph into smaller chunks, each managed by a specific vertex of the graph. The framework also provides a reconfigurable architecture targeting FPGAs. This architecture outperforms CPUs by over 26x and enterprise GPUs by over 4x. Moreover, the proposed partitioning scheme can be applied recursively, graphs can be partitioned into subgraphs, and these subgraphs can be partitioned again. This allows applying the proposed framework to distributed computing. A graph can be portioned and assigned to nodes first, and then these partitions are further split for parallel processing on FPGA accelerators, if available on the node. This study analyses the integration between the proposed framework and Hadoop for distributed computing and proposed forecasts based on the measured performance combined with the well-known scalability characteristics of Hadoop [21, 34].

The main contributions of this paper are as follows:

- Introducing a framework of reconfigurable architecture suitable to process very large-scale graphs. This framework gets the benefit of an offline partitioning scheme to manage the underlying FPGA devices. This work shows the potential of using this approach as an efficient core of a distributed platform;
- Analysing the large-scale graph computing challenges on FPGAs by presenting a baseline study and distribution methodology toward processing large-scale graphs on Data Centre acceleration platforms;
- A novel model based on Hadoop to distribute the graph processing workload on the available workers. This model provides a flexibility to execute a very large scale graph dataset on the available resource either CPU or FPGA which is efficient and cost-effective.
- Proposing an optimised implementation of the PageRank for a single FPGA, which outperforms state-of-the-art open source solutions on CPU, GPU and FPGA offering a speedup up to 2×, 4.4× and 26× higher.

The rest of this paper is structured as follows: in "Problem definition" section, we describe the background and the motivation of this study. In "Existing solutions" sections, we discuss related studies and their features compared to our study, and we introduce a taxonomy of the recent works on FPGAs and their characteristics. Then in "Proposed solution" section, we introduce the proposed solution in "Elaboration" section, and we further discuss the baseline study, the methodology of design implementation and its evaluation. Finally, in "Conclusions" section, we conclude and briefly introduce future works.

## Problem definition

Recent studies on graph processing on FPGA [16, 19, 51] evaluate their work with medium-sized graph datasets instead of using large-scale ones. Hence, these graphs can be computed by a desktop CPU, as shown by Sakr et al. [49]. This may discourage the usage of hardware accelerators, like FPGAs or GPUs since they are generally more costly and less programming friendly than a general-purpose CPU. However, the rise of big data technologies made it easier to collect, store, and process large volumes of data. This led to more data being available to be represented as a graph, resulting in larger graph sizes. In fact, graph size is rapidly growing, reaching the order of Peta Bytes [49], exceeding the main memory storage capacity available on modern CPUs or GPUs. Thus, the motivation of our work is large-scale graphs evaluation, also considered in recent works on FPGAs [6, 19, 51].

The second motivation is integrating a high-level interface to deploy a distributed platform on top of the underlying hardware. Hadoop is a valuable solution for large-scale graph processing because it provides a powerful set of tools for storing, processing, and analysing large graph datasets in a distributed manner. The Hadoop Distributed File System (HDFS) allows for the storage of large data sets across a cluster of machines, making it possible to process graphs that are much larger than what a single machine can handle. The scalability of Hadoop allows the cluster to be easily adapted by adding or removing machines, allowing the processing of large graphs cost-effectively. The map-reduce programming model employed by Hadoop facilitates distributed computing, which can greatly improve the performance of graph processing algorithms.

## Existing solutions

This section analyses alternatives and state-of-the-art approaches to large-scale graph processing.

### Single-FPGA based frameworks

Zhou et al. [69] proposed a system that employs the edge-centric processing model and the GAS (Global Address Space) paradigm to handle medium-sized graphs in a systematic framework. They address the FPGA chip’s memory space limitation by using part of the onboard DRAM for updates. This buffer temporarily stores intermediate processing results, but it creates a significant I/O overhead that lowers graph processing performance.

To enhance pipeline efficiency and graph processing speed, FabGraph is using a 2-level caching mechanism for vertices that periodically stores vertex blocks. However, when extremely sparse real-world graphs are used, streaming processing creates large communication overhead between the two cache levels [51].

FPGP [18] prepares a large input graph using grid blocks and stores the graph’s vertex and data in onboard and host DRAM. During computation, the edges are sent to the FPGA through the host bus and processed.

GridGAS [74] utilises the GridGraph [73] graph partitioning and proposes a method for processing massive graphs using a heterogeneous FPGA accelerator. The graph data is immediately sent to the FPGA chip processing unit. However, the system performance is affected by the PCIe limitation due to the data transfers between the host and the FPGA device. Low bandwidth results in poor processing performance and low utilisation rates of the FPGA chip’s resources.

GraphOps [46] presents a modular Dataflow library approach to build a graph processing accelerator on FPGA written in MAXJ using the Maxeler toolchain. There are some limitations with GraphOps: a lack of portability and memory consistency, also it only supports one FPGA.

GraphGen [45] transforms the input graph into an instruction stream, which is then processed by pipelines implemented using FPGA’s logic resources. The work utilises the DRAM memory interface known as CoRAM, which enables the FPGA to access the host’s main memory. The accelerator is a single processing device that leverages parallelism from the application via pipelining and SIMD processing.

Asiatici and Ienne [6] have introduced large-scale graph processing on a single FPGA by implementing the work proposed in Chisel using the Vivado design suite for the synthesis process [7]. The evaluation was conducted on Amazon AWS F1 instances, which include a Virtex UltraScale+ FPGA connected to the host system through PCI Express and four 16 GB DDR4 channels. The contribution of the work is to eliminate cache misses and exploit the multi-die feature of a single FPGA.

ThunderGP [16] offers an automated user interface for graph processing, enabling users to automate the execution of desired applications. To ensure efficient use of the platform’s memory bandwidth, ThunderGP employs methods to process the appropriate number of kernels while fitting them within the constraints of the device. ThunderGP groups Scatter Processing Elements (PEs), these PEs together in a kernel group known as a “scatter–gather” kernel group, as they operate in the same pipeline. Apply PEs, on the other hand, are placed in a separate kernel group referred to as an apply-kernel group.

#### Multi-FPGA based frameworks

GravF-M [24] provides a redesigned architecture from their previous work [23], that expand the architecture over the distributed platform and aims to minimise communication across the inter-FPGA network. Although network bandwidth is the limiting factor for distributed computing performance on most systems, a proper design can increase overall system compatibility and performance. In GravF-M, authors design a scatter-apply-gather paradigm among multiple PEs, communicating with other processing elements on another FPGA board through the network interconnect. A Processing Element (PE) here is a minimal hardware function that expresses the proposed method. GravF-M also incorporates a low-overhead partitioning technique that improves load balancing among PEs and FPGAs. The FPGA kernel is performed for each active vertex in the graph called *superstep*. A vertex kernel only has access to constrained data locally to the vertex during a superstep. Messages are used to share data with neighbouring vertices. Gather, Apply, and Scatter are three further functions included in this implementation. On the contrary, while we use GridGraph, we do not need to implement these stages since GridGraph has already combined the three phases into one stream-apply phase in that every edge is streamed, and the produced update is promptly applied to the source or destination vertex. Only one traverse across the edge blocks is required by aggregating the updates. This significantly simplifies the design and exploits better parallelism since the processing element can be duplicated in the FPGA resources with much more numbers than presented in GravF-M. The programming model used in GravF-M is Migen, a Python-based tool to export Verilog codes to be synthesised with conventional tools such as Vivado. The important limitation of GravF-M is the limited dataset size, which needs to fit the entire graph in the resources provided by the FPGA. Moreover, the evaluation has been done on just Synthetic graphs like RMAT [14] to make sure the load is balanced through the distribution in the network and the size of the evaluated dataset is small. Another limitation of GravF-M is the compatibility of the work to be extended to large-scale graphs; first, the whole graph must fit onto the FPGA; second, there is no supervision from the host, and orchestration of the work must be considered and hard coded from the initial implementation. Whereas, in our proposal, the host orchestrates the graph’s workload and distributes it among the available FPGAs, which allows for dynamic load balancing and scheduling.

In ForeGraph [19] authors propose a graph processing framework that utilises the onboard DRAM grid representation of graphs and distributes FPGA logic resources within several pipelines. Each pipeline consists of two vertex buffers that preserve vertex blocks. Through these pipelines, the blocks dedicated to each vertex are first loaded into the vertex buffers connected to the buffers, and then the edge blocks are processed in parallel by the FPGA chip. In this technique, the pipelines interface explicitly with the DRAM to swap vertex data, resulting in small pipeline delays and improved graph processing performance.

Although ForeGraph provided a state-of-the-art competitive evaluation against other recent studies, the work is based on simulation, and output results have not been experimentally validated on a real hardware platform. The critical point is that the presented approach in this work is likely to face hardware limitations such as the clock and timing constraints of actual hardware. Additionally, the overhead of the network is neglected since the overhead of such network interconnects is not considered in the evaluated results on a simulated platform, which can make a significant difference.

There are few and limited works that elaborate on Hadoop on multi-FPGA platforms [2, 17, 43]. Neshatpour et al. [43] propose a Hadoop machine-learning system using multi-heterogeneous platforms. This work proposes a system including a master desktop responsible for hosting the Hadoop, and it is connected via a switch to a number of heterogeneous FPGA boards. The authors focus more on profiling the characteristics of the design, such as I/O overhead and kernel movements, to show the potential of the design and speed up by using the Hadoop framework. However, it is not clear and discussed in detail the structure of the design in FPGA and its interface between the heterogeneous platform itself (data movement between PS and PL part in the heterogeneous FPGA board) and the host system.

In [2, 17], the authors proposed a Hadoop cluster framework using FPGA boards to accelerate machine learning applications. In these studies, the goal of the work is to distribute deep computation load into a Hadoop cluster or cloud of computing nodes and use FPGAs to accelerate the intensive computational kernels. The crucial point of these works are; first, there is not a clear design specification to study how the FPGA kernel units interface with the higher-level hosts; second, the observed speed up is not well studied against state-of-the-art and against other studies on CPU or GPU.

FDGLib [60] is a lightweight communication library that facilitates the scaling out of single FPGA-based graph accelerators to a distributed version in a distributed platform with minimal hardware engineering efforts. To make any graph suitable for this method, the library provides APIs based on Message Passing Interface (MPI) that can be integrated into FPGA-based graph accelerators with minimal modifications to their existing code. One of the critical aspects of FDGLib is the substantial preprocessing time overhead. According to the research paper, the preprocessing time for the smallest evaluated dataset can take up to 50 s, while in our framework, the preprocessing time is in order of a few milliseconds to a few seconds, which has a very low impact on the evaluation of the work described in "Evaluation" section. Furthermore, it should be noted that the dataset used in the study is relatively small, and as such, the preprocessing time may have a more significant impact on larger datasets.

Table 1 shows a taxonomy of the selected best existing solutions that are closest to our work.

**Table 1:** Brief overview of the closest recent studies on FPGA accelerators and their features compared to this work

Work	Distributed?^a^	Language^b^	Implementation^c^	Access to host memory^d^	Evaluation size^e^	Public repository?^f^	FPGA platform^g^	Published year^h^
ForeGraph [19]	$\checkmark$	HDL	Simulation	$\times$	Medium	$\times$	Xilinx VCU110	2017
FabGraph [51]	$\times$	HLS	Simulation	$\times$	Medium	$\times$	Xilinx VCU110 and VCU118	2019
HitGraph [70]	$\times$	HDL	Hardware	$\times$	Small	$\checkmark$	Xilinx Virtex Ultrascale+	2019
ThunderGP [16]	$\times$	HLS/C++	Hardware	$\times$	Medium	$\checkmark$	Alveo Family	2021
GraVF-M [24]	$\checkmark$	Python^i^^i^	Hardware	$\checkmark$	Medium	$\checkmark$	Micron Pico se-6 platform	2019
GridGAS [74]	$\checkmark$	HDL	Hardware	$\checkmark$	Medium	$\times$	Xilinx Kintex	2018
FPGP [18]	$\times$	HDL	Hardware	$\times$	Medium	$\times$	Xilinx Virtex-7	2016
FDGLib [60]	$\checkmark$	HDL/C++	Hardware	$\times$	Small	$\times$	Alveo Family	2021
Asitatici and Ienne [6]	$\times$	Chisel	Hardware	$\times$	Large	$\checkmark$	Xilinx Virtex Ultrascale+ (AWS Platform)	2021
GraphOps [46]	$\times$	MAXJ	Hardware	$\times$	Small	$\times$	MAXELER Boards	2016
**This Work**	$\checkmark$	HLS/C++	Hardware	$\checkmark$	Very Large	$\checkmark$^j^	Alveo Family	2022

^a^ Weather the algorithm supports distributed computing

^b^ The programming language used

^c^ Weather the implementation is based on software simulation or actual hardware

^d^ Weather the hardware access to the Host Memory directly

^e^ The scale of the evaluation graph presented dataset. Here, we consider a graph with 10 GB data size as a Medium size graph

^f^ Weather the work is open-source and available to the public

^g^ The target platform of FPGAs discussed in the paper

^h^ The year the work was published

^i^ Migen, a Python-based tool to export Verilog codes to be synthesised with conventional tools such as Vivado

^j^ The source code is available at: https://github.com/AminSahebi/distributed-graph-fpga.git

## Proposed solution

When implementing a large-size graph processor on FPGAs, there are several design choices to consider. Firstly, due to the limited on-chip memory available on modern FPGAs, it is necessary to partition the graph into small chunks that can fit into it. This partitioning method must also produce equally sized chunks, as FPGAs do not support dynamic memory allocations. Moreover, the partitioning scheme should minimise external memory accesses as data transfers introduce a huge amount of overhead and kill the performance. Secondly, there is a need to design a processing kernel that has a memory access pattern compatible with the partitioning scheme mentioned above. This allows for a reduction of the communication overhead as communications are costly, especially in case host computer memory needs to be read from the accelerator or different accelerators. The processing kernel should use the least amount of resources. On FPGAs, parallelism is achieved in space by assigning different computational resources to a different portion of the workload. Computational units can be instantiated multiple times until there are no resources available. Kernels that use fewer resources can be instantiated more often, increasing parallelism and performance. The partitioning scheme being discussed in this study is designed to split the workload among different nodes. The scheme is similar to the partitioning scheme required for FPGAs. It is applied recursively, once to partition the graph among different nodes and again to map the subgraphs to on-chip accelerator memory. This approach allows for efficient and effective use of resources in distributed and parallel computing environments.

The following section describes the approaches used for the partitioning method, the single-FPGA and multi-FPGA implementation.

### Graph partitioning

A common challenge discussed in the graph computing literature is graph partitioning. Many works proposed novel techniques and algorithms for graph partitioning [31, 37, 73]. Table 2 shows the best graph partitioning presented in recent studies.

The proposed work involves the GridGraph partition method for dividing the edges of a graph into smaller groups, called chunks and assigning each group to a specific vertex. The chunks and their corresponding vertex information are then stored on the host file system. The chunks should be independent of one another, and their size should be compatible with the size of BRAM on the target FPGA. The Kernel will read the chunks sequentially from the host memory, and updated values will be written back to the host memory.

**Fig. 1:**
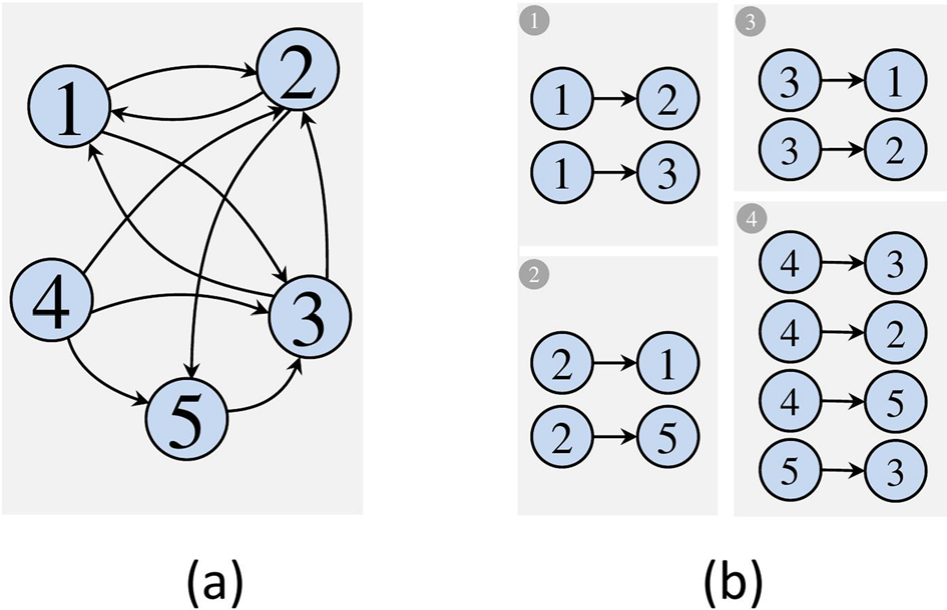
**a** A given sample graph. **b** Edge blocks results of preprocessing concept [48]. The ❶, ❷, ❸ and ❹ are referred to as the produced edge blocks using GridGraph partitioning [73]. Here, the number of partitions is P = 2, producing $P^2$ edge blocks

Among the various partitioning techniques present in the literature (see Table 2), GridGraph offers the best tradeoffs for FPGA acceleration. Hence, the GridGraph partitioning method is selected due to its ability to offer high data locality and avoid data conflicts. This is achieved by dividing the graph into smaller subgraphs called grids, processing each grid independently, making it more efficient when large graph processing is considered [73].

Grids can be mapped on the on-chip resources on the FPGA, improving the performance scalability.

An example of graph partitioning with GridGraph is illustrated in Fig. 1, whose vertex set is divided into two partitions (P), resulting in four equal-length 2x2 grid subsets. It can be seen that a given directed graph G = (V, E), where V indicates the set of vertices and E the set of edges^1^, will be divided into $\mathrm {P^2}$ blocks based on the source and destination vertices. Each edge is placed into a block using the following rule: “*the source vertex determines the row of the block, and the destination vertex determines the column of the block.*” Each partition represents outgoing edges for a range of vertices; partition P_1_ holds outgoing edges for vertex 1, P_2_ holds for vertex 2, P_3_ holds for vertex 3, and P_4_ for vertices 4 and 5.

The graph is processed by the iterative process in a predetermined sequence. Specifically, it loads edges from partition P_1_ and processes them in memory, followed by loading edges from partition P_2_ and so on until the last partition P_4_ is processed. After all the partitions have been processed, the process computes vertex values that may be stored on disk to end the iteration. This process is repeated for multiple iterations until a termination condition specific to the algorithm is met. Further details about the implementation will be presented in the subsequent sections.

**Table 2:** Most recent and well-known graph partitioning techniques suitable for FPGA implementation

Graph partitioning algorithm	Methodology	Programming Language	Graph partitioning	Source code	Platform	Year
GridGraph [73]	Grid partition of edges	C++	Store edge partition blocks on disk	Public	CPU	2015
Lumos [57]	Grid partition of edges plus cross-iteration propagation values support bulk synchronous processing	C++	Store edge partitions as blocks on disk	Public	CPU	2020
FabGraph [51]	Grid partition of edges plus hash partitioning to support power law graphs	C++	Store partition blocks on disk	not public	Multi-FPGA	2019
PowerGraph [31]	Vertex-cut partitioning	C++, Java, Scala	Partitioning during Runtime	Public	CPU	2013
Graphchi [37]	Shard-interval partitioning plus sorting, asynchronous processing	HLS	Partitioning during Runtime	Public	Multi-FPGA	2021
ThunderGP [16]	Vertex-cut partitioning	HLS-C/C++	Partitioning during Runtime	Public	single-FPGA	2021
Foregraph [19]	Shard-interval	HDL	Partitioning during Runtime	not Public	Multi-FPGA	2017

### Single-FPGA graph processing

In a single FPGA workflow, a graph dataset is pre-processed using the partitioning method described in the "Graph partitioning" section, and the resulting edge blocks are stored on the host file system. The overall size of graph blocks may exceed terabytes, and the host memory must be enough to read all these edge blocks from the file system. The data is loaded into the FPGA’s on-chip memory before it can be processed. Dedicated FPGA kernels read the data from the stream input provided by the host and direct it to the FPGA computational units. Once the computation is complete, the aggregated results are written back to the host memory and stored in its file system.

**Fig. 2:**
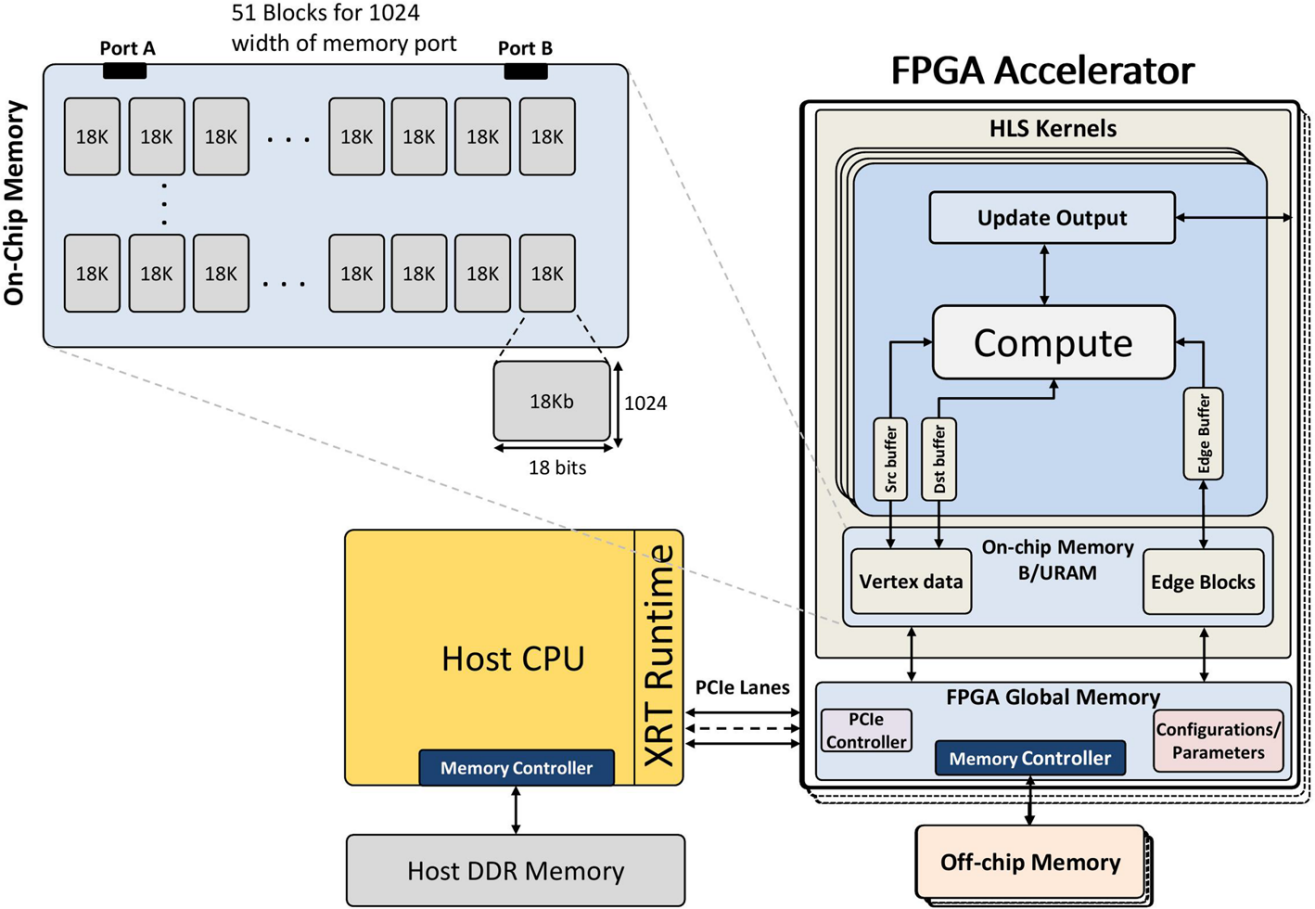
The design system overview of the single-FPGA processing unit

Figure 2 shows an overview of the single FPGA graph processing unit. In this figure, on-chip memory is configured to achieve the highest memory bandwidth while keeping the frequency of the system at its highest.

### Multi-FPGA graph processing

Large graph datasets can easily exceed the computing capabilities of a single machine (e.g., memory). In distributed computing, data is split among multiple machines, enabling the processing of graphs that are beyond the capabilities of a single machine. Distributed systems can provide several benefits, including scalability, fault tolerance, and performance. A distributed system consists of a set of machines working together as a single virtual system, with each machine or node responsible for processing a portion of the data. These systems can be manually managed with a custom software implementation, for example by leveraging the widely used message passing interface (MPI) [59]. Also managing these systems manually allows for fine-tuning the application and enables achieving high performance. This requires significant engineering effort and expert developers in the context of distributed computing.

Moreover, custom solutions offer no guarantee in terms of scalability and performance.

There are a wide variety of frameworks for distributed computing that automate and overcome most of the challenges mentioned above. Hadoop is a popular open-source framework that is used for large datasets on clusters of machines. It was created by the Apache Software Foundation in 2005, and has since become one of the well-known used technologies for large data processing [5]. Hadoop is based on the map-reduce programming model, which allows for the parallel processing of big data across a distributed platform thanks to the adoption of the Hadoop Distributed File System (HDFS).

The data is usually stored in a distributed file system, such as Hadoop Distributed File System (HDFS) or ZFS [47] and processed using a distributed computing framework (e.g., Apache Hadoop). Graph algorithms, such as PageRank, can be implemented on top of these frameworks to process and analyse the graph [22]. The use of FPGAs in combination with Hadoop for large graph processing is an emerging field that has gained attention in recent years [11, 53]. By combining Hadoop with FPGAs, it is possible to take advantage of the scalability and fault-tolerance of Hadoop, while also leveraging the high performance of FPGAs for graph processing tasks. Adoption of FPGA with Hadoop is still in the early stages, and more research is needed to analyse the feasibility of using FPGA combined with Hadoop to accelerate graph processing and optimise the performance of these systems.

**Fig. 3:**
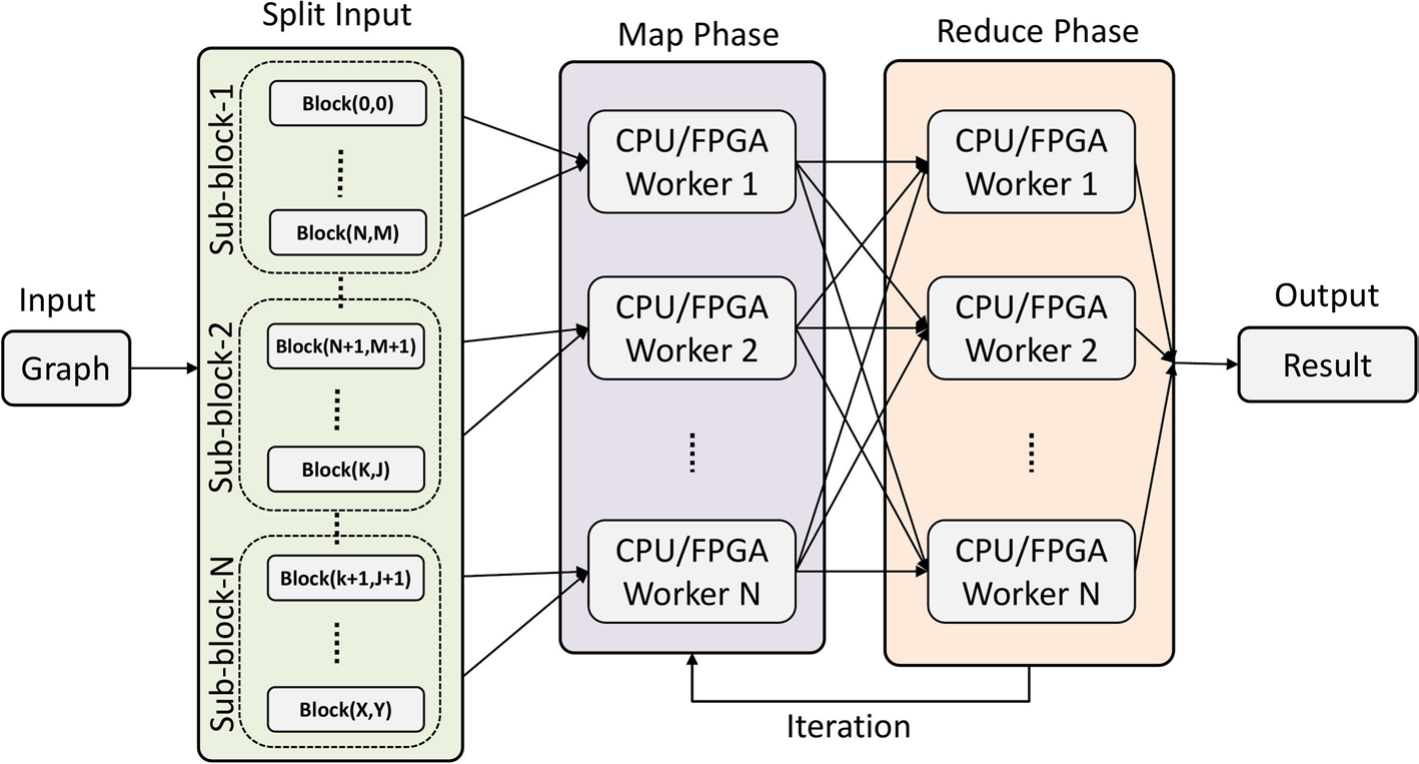
The Hadoop framework for distributed graph processing [48]

Data processing can be divided into two main phases:

1. The first phase is known as the “Map” phase. The data is divided into smaller chunks, called input splits, and each split is processed by a separate node in the cluster. The processing that occurs in the Map phase is typically performed by user-defined functions called Mappers, which take the input data and transform it into a set of intermediate key-value pairs;
2. The second phase is known as the “Reduce” phase. The intermediate key-value pairs from the Map phase are processed by user-defined functions called “Reducers”, which take the input data and merge it into the final output.

In a map-reduce architecture, a user application launches a root controller and a set of mappers and reducers, which are distributed across several compute nodes. The root node coordinates the generation of mappers and reducers and keeps track of their progress. The overall system overview of the Hadoop map-reduce design is illustrated in Fig. 3.

In our case, nodes containing more than one FPGA accelerator are configured in a way that Hadoop sees them as multiple nodes with a single FPGA. For example, on a node containing four FPGAs, four different instances of Hadoop are executed, and the FPGAs are mapped one-to-one to the instances. This design choice greatly simplifies the design as it removes the need to split the workload between multiple FPGAs manually. Additionally, it enables the use of Hadoop scheduling for load balancing and fault tolerance. The Hadoop scheduler can then handle single FPGA failures without switching offline the entire node.

**Fig. 4:**
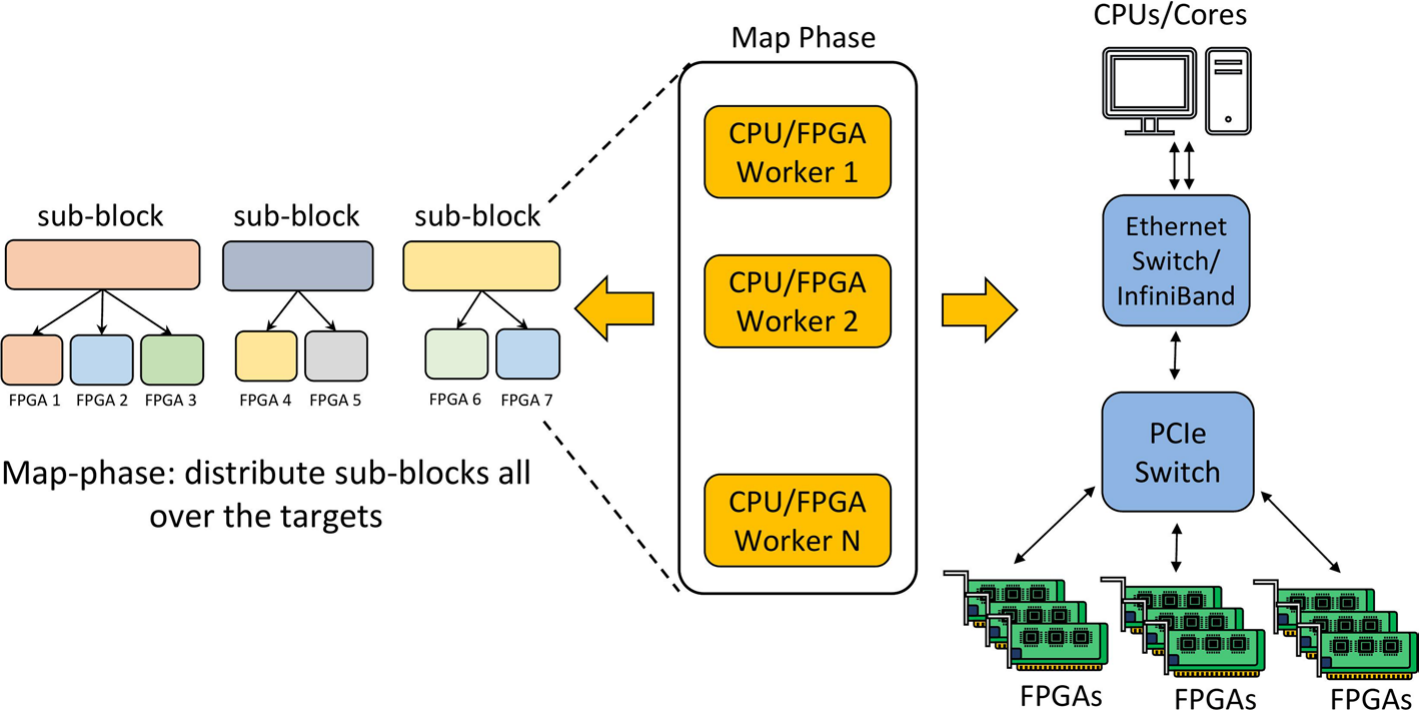
Map-phase, distribute sub-blocks all over the targets

**Fig. 5:**
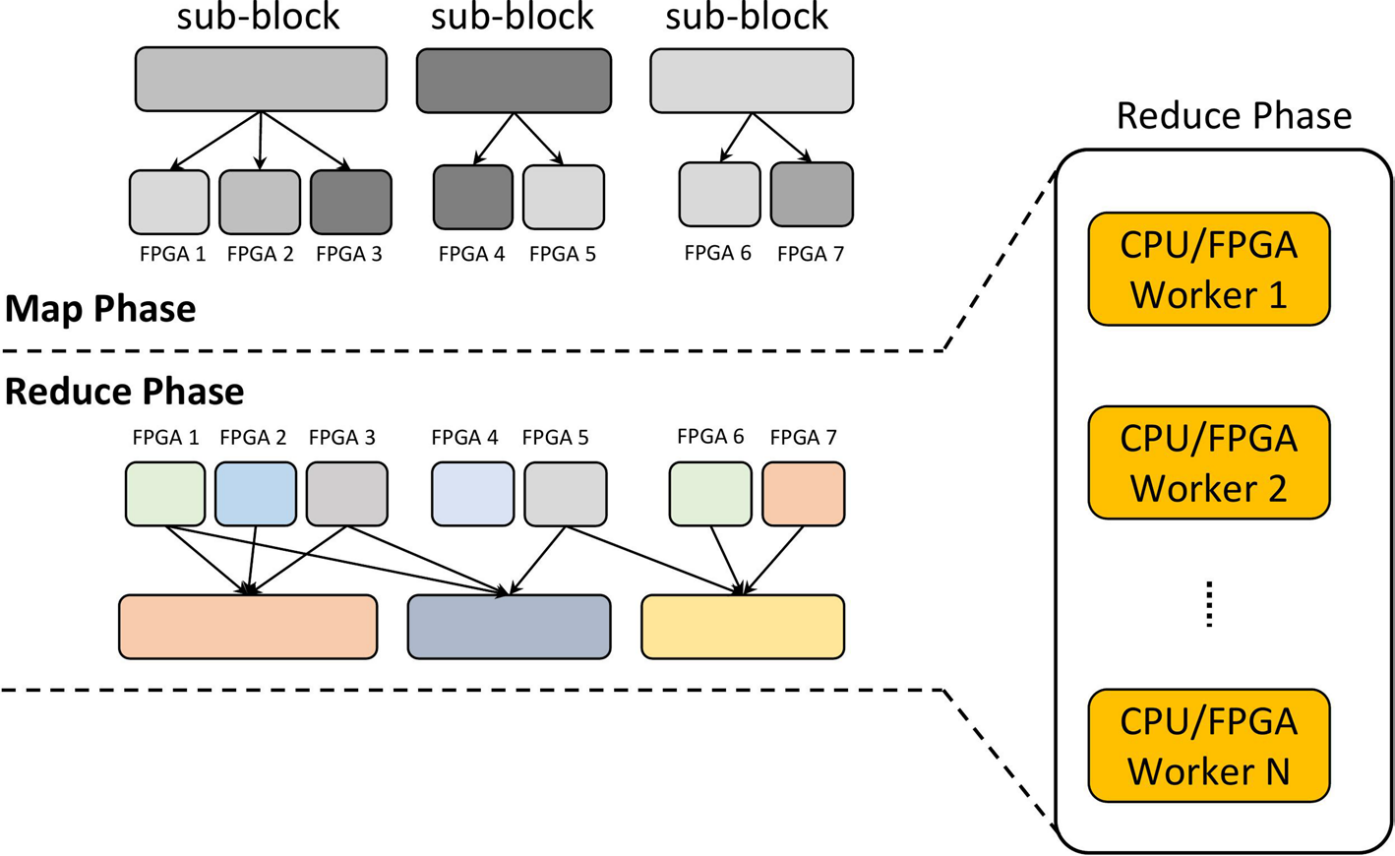
Reduce-phase, gather partial workers’ results to compute the final output

The proposed large graph processing framework involves three phases:

1. Split the graph into sub-blocks by using the proposed variant of the GridGraph partitioning method (e.g., edge blocks);
2. Execute parallel portion of the graph processing algorithm implementing a custom *Map* function (see Fig. 4);
3. Merge the partial results computed on different workers using a custom *Reduce* function (see Fig. 5).

Phase 1 can be considered pre-processing, where the graph is partitioned into sub-graphs, and the various node will process them. This process can be executed in parallel and consists of a single scan of the graph.

Phase 2 is part of the actual processing. This step consists of executing the graph processing algorithm on the subgraphs computed in the previous step. This step is implemented using a custom *Map* function. In the proposed architecture, this step computes the PageRank of the subgraph by evaluating the rank equation.

Phase 3 is the final step of the processing. It consists of merging the partial rank vectors into one. In the proposed architecture, this step is optional. Since Mappers save the results on different files, this step can be used to merge those files into one. However, depending on the use case, merging the results into one monolithic file might not be needed.

Phase 1 is repeated two times, the first time to split the graph into subgraphs that are mapped to nodes and the second time to further split the subgraph into chunks that can be processed in parallel by the FPGAs. More details about this can be found in "Single-FPGA graph processing" section. In the case of iterative algorithms, such as PageRank, phases 2 and 3 need to be executed multiple times until the results converge.

## Elaboration

To evaluate the proposed framework, the following steps are taken.

Initially, we defined a theoretical performance model to understand whether or not some advantage could have been achieved by deploying FPGAs for accelerating part of the computation. The model is first evaluated by using maximum values for the basic parameters (e.g., maximum bandwidth for memory, see Table 3). The performance model evaluation is conducted by using selected datasets, which are large enough in terms of vertices and edges to identify any potential limitations or issues (see Table 5). Since FPGA programming requires significant engineering effort, modelling the performance using conservative worst-case scenario parameters is necessary to determine the trade-off between development time and achievable performance [58]. Then, in "FPGA implementation" section, the methodology adopted to implement optimised graph algorithms (e.g., PageRank) on cloud-based FPGA devices (e.g., Xilinx Alveo Boards) is described. Lastly, the performance of the optimised implementation is compared with CPU, GPU, and FPGA solutions, and distributed system forecasts are provided (see "Evaluation" section).

### Methodology and baseline

FPGAs are inherently hard to program. Present-day HLS toolchains simplify this process. However, deploying an application on FPGA still requires significant engineering effort. Engineers developed a method to quickly predict the performance of FPGAs and determine if they can meet the necessary requirements, reducing wasted time and effort [58]. In this study, we employ this methodology to guide the FPGA development process. The exact details of this methodology are not part of this study, but the main steps are briefly summarised. The first step involves analysing the algorithm and drafting a possible FPGA architecture. This architecture is then analysed, accounting for input size, data transfers, the bandwidth of the interconnects and accelerator characteristics. FPGA performance is predictable and can be estimated “a priori” by analysing the workload and using a set of linear equations [58]. This performance forecast is then evaluated, and in case the performance requirements are not met, the previous steps are repeated multiple times with new and improved FPGA architecture candidates. Once an FPGA architecture candidate is found, there is a need to produce a software model that simulates the FPGA architecture exactly. Using metrics from the software model, the performance forecasts are refined, and if needed, the architecture is changed accordingly. For the sake of clarity, the various intermediate architectures that are developed and refined at each iteration are omitted. Only the final version is described. The assumption and notations used in the baseline model are presented in Table 3. The model is general and can be applied to any FPGA by inputting the correct parameters. However, the results show the baseline only for our selected FPGA accelerator, as it is the FPGA device available for this study (see "FPGA implementation" section). The most important formulas used in this forecast are summarised in Table 4. Some formulas related to the data partitioning are extracted from the work presented in [73].

Tables 6, 7, and 8 show the performance based on the system characteristics and assume the use of Xilinx Alveo U250 in our performance model. As can be seen, the anticipated speedup is calculated based on the computation time, communication time, and other overheads and bottlenecks such as PCIe Rate. The efficiency of the PCIe bus is set to 0.85 of its ideal performance. This is a worst-case scenario, where the application, encoding and packaging of data reduce the bandwidth by 15%.

**Table 3:** Baseline assumption and notations used in the baseline study

Symbol	Meaning	Con-stant value(if any)
$\left|V\right|$	Number of vertices	
$\left|E\right|$	Number of edges	
S_v_	Size of a vertex (Byte)	4
S_e_	Size of an edge (Byte)	8
data_width_	Depending on the scenario (1024, 2048, 4096)	2048
$P^{2}$	The graph will be portioned into P$\times$P edge-blocks	
S_block_	Size of an edge-block (MB)	10
f_FPGA_	Clock Frequency MHz	200
T_comp_	Time to process (read/Write to/from-chip BRAM)	
T_comm_	Time to move data from host memory to FPGA	
T_init_	Time to load the libraries (reduced after the first run)	
T_PCIe_	Time to interconnect between boards	
T_mem_	Time to transfer data (loading blocks) between off-chip memory and PEs	
T_baseline_	Time for CPU-only	
T_FPGA_	Execution time in FPGA only	
S_bram_	BRAM storage size [it should be higher or equal to the size of each edge block Sbram ≥ Sblock(i,j)] (MB)	54
BW_mem_	DDR4-2400 (GB/s)	19.2
BW_PCIe_	PCIe 3.0 bandwidth ×16 (GB/s)	32
eff_PCIe_	PCIe efficiency	0.8
eff_mem_	Memory efficiency	0.8
SL	Number of SLRs in the FPGA die	4

**Table 4:** Formulas used in the baseline study

Formula	Description
$\text {P}=\left| \sqrt{\frac{S_{e}\times \left| E \right| }{S_{block}}} \right|$	Total number of partitions based on GridGraph preprocessing
$Size_{data} = S_{block} \times {P^2}$	Edge block size to be processed
$BW_{BRAM} = \frac{data_{width} \times {f_{FPGA}}}{S_{e}}$	BRAM Bandwidth calculation
$T_{mem} =\frac{\left| V \right| \cdot S_{v} + \left| E \right| \cdot S_{e}}{BW_{mem} \times {eff_{mem}}}$	Load time for Vertex and Edges data from/to Global Memory to/from PE_FPGA_
$T_{PCIe} = \frac{size_{data}}{BW_{PCIe}\times {eff_{PCIe}}}$	PCIe transfer time
$T_{comm} = T_{init} + T_{PCIe} + T_{mem}$	Transfer time from/to CPU host to/from PE_FPGA_
$T_{FPGA} = T_{comm} + T_{comp}$	Total time for FPGA
$T_{comp} = \frac{\left| V \right| + \left| E \right| }{BW_{BRAM}}$	Computation time in the FPGA
$S = \frac{S_{baseline}}{S_{FPGA}}$	Speedup over the baseline execution time

Table 6 shows the loading time from the host memory to the FPGA device’s global memory. Here, T_mem_ is evaluated on the graph data and the maximum performance of the DDR4-2400, which is installed on the host system.

**Table 5:** The datasets for evaluating our proposed study

Graph dataset	Vertices	Edges	Size (GB)	Type
LiveJournal [38]	4.8M	0.069 B	1.1	Social web
Web-UK-2005 [12]	39M	0.994 B	16	Web graph
Twitter [36]	41.6 M	1.47 B	23	Web graph
Friendster [38]	68.3M	2.58 B	43	Social web

We choose them based on the size and the structure of the datasets to be comparable with other works

**Table 6:** Load time for vertices and edges data from global memory to FPGA-PEs (efficiency = 0.8)

Dataset	Vertices	Edges	S_v_ (bytes)	S_e_ (bytes)	T_mem_ (s)	BW_Mem_ (GB/s)
LiveJournal	4.85 M	0.069 B	4	8	0.0372	19.2
Web-UK-2005	39.1 M	0.994 B	4	8	0.49	19.2
Twitter	42.1 M	1.47 B	4	8	0.776	19.2
Friendster	68.3 M	2.58 B	4	8	1.36	19.2

Table 7 shows the computation and communication time that needs to be done from the host to the FPGA device. These results are derived from the previous Table 6. Note that data is assumed to be already loaded in the memory of the host system; the time to read data from the host file system is omitted.

**Table 7:** Computation and Communication Time from CPU host to FPGA

Dataset	PCIe-3 16-lane data rate (GB/s)	Data Size (GB)	T_PCIe_ (s)	T_init_ (s)	T_comm_ (s)	T_comp_ (s)
LiveJournal	32	0.526	0.0244	0.1	0.16	1.76
Web-UK-2005	32	7.2	0.27	0.1	0.87	23.2
Twitter	32	11.2	0.44	0.1	1.32	36.00
Friendster	32	20.2	0.7	0.1	2.23	101

Finally, Table 8 shows the overall speedup against the same algorithm on the CPU. The values presented in this table are derived from the two previous Tables 6 and 7 discussed in this section. The number of partitions is assumed to be constant in all graph datasets, and the number of Super Logic Regions (SLRs) for this evaluation is assumed to be one. SLRs are specific areas of the FPGA fabric that can be used to implement logic functions or memory elements. By allocating each kernel to a specific SLR and dedicating the necessary memory channel, the design can be optimised and the scalability improved. The baseline shows transferring edge blocks with bigger sizes is better compared to the CPU baseline, and this is the potential of the design, which is suitable for very big graph datasets. Based on this performance evaluation, we achieved close to $\sim$13 times (Friendster dataset) more than the baseline implementation on the CPU.

In other words, our model shows that FPGAs have the potential to be one order of magnitude faster than CPUs for graph processing. Hence, the proposed architecture is developed and implemented onto FPGA. The following "FPGA implementation" section shows technical details of the implementation.

**Table 8:** The performance model report, which has been calculated based on the bottlenecks like PCIe Rate, Computation time, Communication time, etc. Here the number of Super Logic Region (SLR) used is equal to 1

Dataset	Partitions	SLRs	T_FPGA_ (s)	T_baseline_ (s)	Speedup
LiveJournal	16	1	1.92	12.86	6.7
Web-UK-2005	16	1	24.14	270	9.9
Twitter	16	1	37.3	538.1	7.2
Friendster	16	1	197.8	1340	12.6

### FPGA implementation

The baseline study presented in the previous section demonstrated the advantages of using FPGAs for graph processing. In this section, the goal is to present the implementation details and optimisations used to develop the final graph processing framework. Vivado HLS toolchain (version 2022.2) is used for the algorithm implementation on the available Xilinx Alveo U250 board, which contains a Xilinx VU13P FPGA.

**Fig. 6:**
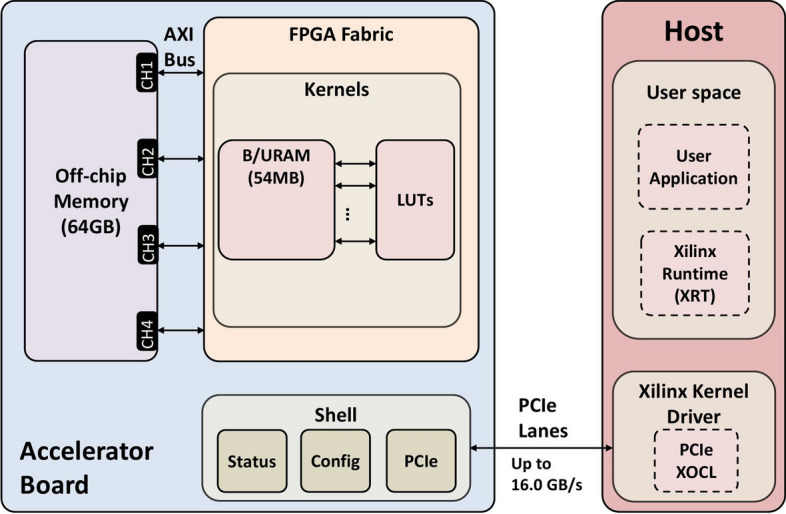
Alveo U250 accelerator card. Some parts are skipped for readability

Xilinx Alveo boards are Data Center accelerator cards that are specifically designed to meet the evolving needs of modern Data Centers, including machine learning inference, video transcoding, and database search and analytics [56]. Figure 6 illustrates the Xilinx Alveo U250 block diagram and its interface with the host system. The Vivado design suite (Vitis version 2022.2) is used to develop and implement the target architecture. The implementation is divided into two parts: *Kernel* implementation and *Host* program. The host code is responsible for the software-based part of the design, which will be executed on the CPU. This part resides on the host system and will schedule the loading of the edge blocks, transfer them to the associated kernels, and, finally, drive kernel execution. Moreover, the host program is responsible for managing the Xilinx Runtime interface (XRT) and programming the bitstream, that is, programming the FPGA to carry out the required computation.

On the other hand, kernel implementation is the part of the application that is executed by the FPGA. The kernel is written in HLS and designed to optimally uses accelerator resources for the best performance. In this study, the kernels implement the graph processing algorithm. Host code and kernels are compiled with a V++ compiler (Vitis C++ compiler with the -O2 optimisation flag).

The following sections describe in detail the Host program, the Kernel implementation, and the optimisation techniques used for the hardware implementation.

#### Host program

The host program can be divided into two main sections.

The first section is to receive the graph and prepare the pre-processed graph data and their parameters. These include the graph block path, the number of vertices, the number of edges, the number of partitions, and edge blocks. Once the graph information is loaded into the host memory, the host program starts to further process data and fetches blocks from the disk to the host RAM. This procedure is out of the evaluation time measurement zone. This methodology is also used in previous studies such as [16, 51].

The next step is to create buffers to keep aligned all data in the memory. This is required to achieve high performance, as the runtime system (XRT) complains about memory misalignment. However, this adds extra “memcpy” during the runtime leading to a longer execution time.

After creating an aligned vector and fetching all necessary information of edge blocks in the Host memory, the bridge between the host and kernels needs to be created. This bridge consists of runtime buffers and commands to program the FPGA device with the bitstream. Finally, data exchange between Host Memory and Kernel local memory (e.g., BRAM) is implemented using OpenCL functions and specific FPGA kernels.

The last step is to instantiate and configure the kernels for the execution within the host code orchestration.

#### FPGA kernels

The efficiency of the FPGA implementation is primarily affected by the use of structures like local arrays. These memory elements are used to store data for a specific function or operation in FPGA. The implementation of local arrays requires utilising memory resources in terms of Lookup Tables (LUTs), Block RAM (BRAM), and registers. However, we needed to carefully allocate local arrays to utilise the resources provided by the FPGA fully.

One way to minimise resource utilisation (e.g., BRAM) and accommodate big local arrays on FPGAs is by partitioning the local arrays and streaming data through small and fast FIFOs (“DATAFLOW” and “PARTITION” keywords).

**Fig. 7:**
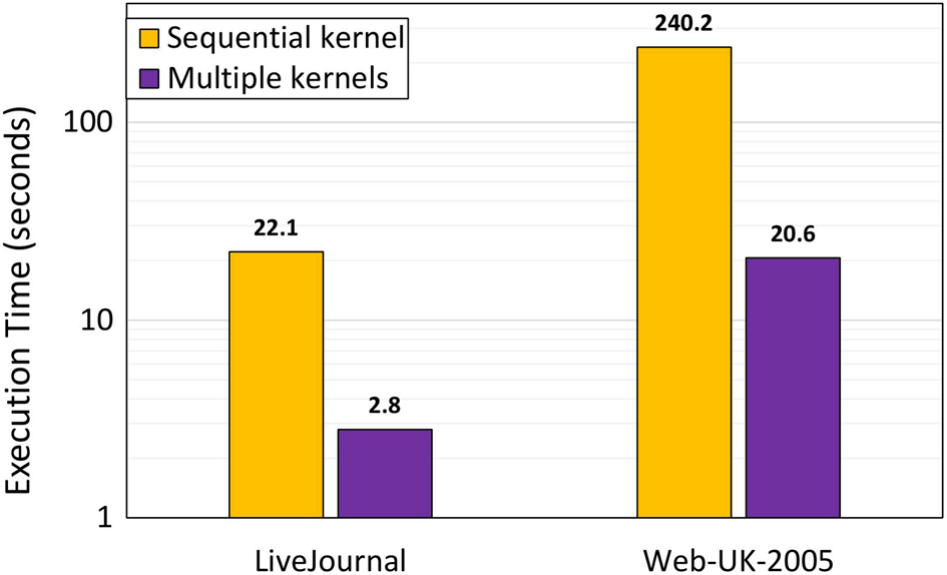
The scalability of the proposed design

The Vitis compiler builds a single hardware instance from a kernel implementation. If the host program executes the same kernel multiple times due to data processing requirements, it must do so on the FPGA accelerator sequentially. The order in which kernels are executed has an influence on overall application performance. In the proposed design, the kernel linking stage is tuned so that a single kernel can instantiate several hardware compute units (CUs). The host program makes several overlapping kernel calls, executing kernels concurrently by running independent compute units, which increases the performance. In the connectivity configuration of Alveo Boards, the allocation of kernels to specific SLRs of the FPGA and the allocation of memory channels is performed in order to optimise the design and improve the scalability. Although the PCIe interface of the Alveo boards has 16 lanes, only 15 lanes can be connected to the kernel instances so that they can communicate with the host simultaneously (this is also confirmed by the official documentation since we must keep one lane for the XRT runtime). Exploiting multiple PCIe lanes significantly optimises the design and the scalability, resulting in a close to 7× speedup when using multiple kernels compared to a single sequential kernel execution (see Fig. 7).

#### Hadoop performance model

In this study, we just focused on the FPGA acceleration rather than the Hadoop scalability details, as the map-reduce programming model has been extensively studied in the literature, and its performance is modelled and predictable [21, 34]. Thus, for the purpose of this study, we forecast the performance by leveraging previous studies that analyse the efficiency of algorithms implemented in map-reduce.

Before starting the analysis, it is necessary to highlight some assumptions:

- The graph is stored on the Hadoop Distributed File System (HDFS);
- The graph is partitioned in parallel by a MapReduce job or custom partitioner.

The first assumption is valid as we target large-scale graphs, which would be far too large to be fit in a single node, hence it is likely that they are already stored in a distributed file system. The second assumption is valid as long as the first assumption is valid. Once the graph is placed on the file system, any pre-processing could be done directly in MapReduce.

Given the previous assumptions, the scale of the graph requires a distributed system to be analysed as a single parallel node would require an unrealistic amount of time. In this study, we analyse the benefit of adding FPGA acceleration to an existing cluster, and the comparison is between a Hadoop cluster with and without FPGA accelerators.

In our proposed architecture, FPGA accelerators are used only in mappers to compute a partial state. The above formula is needed only to account for how a faster mapper will impact the total computation time.

As mentioned above, a Hadoop cluster is not available for this study, and we cannot provide experimental results. Thus, we only provide forecasts based on Hadoop performance models’ algorithmic complexity and link-speed assumptions [21, 34].

The users that already execute distributed graph processing on a Hadoop cluster could use the following formula (Eq. 1) to obtain more accurate performance forecasts by substituting profiler results:

1 $$\begin{aligned} T = T_{split-input} + T_{overhead} + N(T_{scatter} + max(T_{map}) + T_{transfer} + T_{reduce}) \end{aligned}$$

where: $T_{split-input}$ time needed to partition the graph in sub-graphs; *N* is the number of iterations of the iterative algorithm; $T_{overhead}$ is the overhead introduced by map-reduce; $T_{scatter}$ is the time needed to distribute the state vector to all the workers; $max(T_{map})$ is the time needed by the slowest mapper; $T_{transfer}$ is the time needed to transfer the state vector to the reducers (account for shuffle and sort); $T_{reduce}$ is the time needed to aggregate the partial state vectors.

### Evaluation

This section presents the results of the optimised version of the PageRank algorithm for large-scale graph processing using cloud-based FPGA accelerators. In large graph processing, PageRank is often used as a benchmark to evaluate the performance of different graph processing algorithms and systems. PageRank is one of the most computationally intensive graph algorithms, and it requires processing a large number of vertices and edges. PageRank is an algorithm used to evaluate the importance of each vertex in a graph. It has been initially used by commercial search engines to rank web pages by their importance. The algorithm assigns a score, called the PageRank score, to each vertex in the graph. The score is determined based on the number and importance of the vertices that point to it. Vertices with a higher PageRank score are considered more important than those with a lower score. By using PageRank as a benchmark, it is possible to measure the performance of the proposed model in terms of its ability to handle large-scale graph data efficiently.

**Table 9:** The XACC server is used to evaluate the real implementation

Instance Name	CPU	CPU Freq	Cores	Memory	FPGA board
alveo1.ethz.ch	2× Intel Xeon Gold 6234	3.30 GHz	16	376 GiB	Alveo U250

We compare the performance of the proposed implementation of the PageRank algorithm, presented in "FPGA implementation" section, against CPU and GPU and FPGA architectures. The real hardware implementation has been done on a Xilinx Alveo U250 from the Xilinx Adaptive Compute Clusters (XACC) [3]. Each XACC server equally distributes resources into several Virtual Machines (VM) such that each VM has access to one FPGA card. The VM software environment is based on Ubuntu 20.04 and includes software frameworks for FPGA accelerator deployment (e.g., Vitis, Vivado HLS). Table 9 reports the specification of the server and hardware accelerator specifications. For the CPU comparison with PageRank, we used a sequential version, an OpenMP multicore version [15], and GridGraph library [32] as it is one of the most efficient graph processing frameworks on the CPU. The OpenMP version for this evaluation is version 4.0.3, and GCC version 9.4.0 is used to compile the software. Regarding the GPU implementation, we used the cuGraph library [25] for comparison with the CUDA toolkit version 11.7. “cuGraph” is an open-source GPU graph analytics library that is built on top of the RAPIDS ecosystem [25]. It provides a high-performance, easy-to-use, and extensible framework for graph analysis on GPUs. In our experiments, we used the NVIDIA V100 GPU, which is a high-performance GPU based on the Volta architecture.

We selected four different sizes of graphs from the datasets listed in Table 5, from a small size graph (LiveJournal) to a large size one (Friendster), to evaluate our optimised version of the PageRank algorithm on a single FPGA. These datasets were chosen to evaluate the performance when the size of the graph grows to sizes that exceed the memory capacity of the CPU or GPU device memories.

**Fig. 8:**
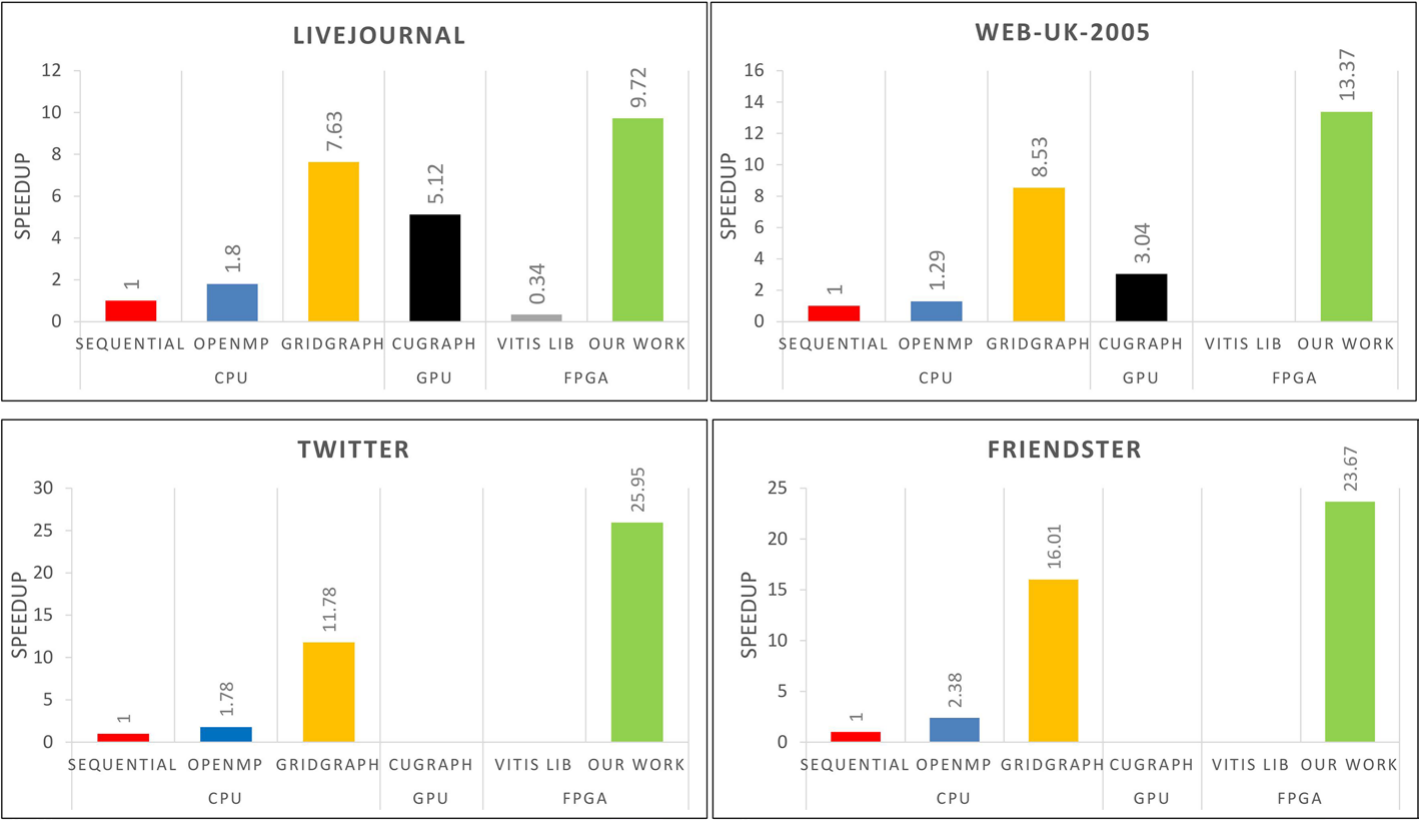
Speedup evaluation of the proposed FPGA PageRank algorithm (our work) with the CPU, GPU, and FPGA solutions for the LiveJournal, Web-UK-2005, Twitter and Friendster datasets

Figure 8 shows that our proposed FPGA implementation of PageRank significantly outperforms the sequential and OpenMP execution on CPU for all of the datasets. The speedup achieved with CPU ranges from about 9.7x with the smallest dataset up to about 26x in the case of the Twitter dataset.

**Table 10:** Alveo U250 platform experimental results in comparison to a software baseline and state-of-the-art FPGA works. Reported numbers are all in Seconds

Dataset	Sequential (CPU^a^)	OpenMP (CPU^a^)	GridGraph (CPU^a^)	cuGraph (GPU^b^)	Vitis Library (FPGA)	Our work (FPGA)
LiveJournal	27.01	5.49	3.54	5.28	79.79	2.78
Web-UK-2005	275.44	185.4	34.9	90.73	N/A^c^	20.6
Twitter	1443	658.5	88.5	Failed^d^	N/A^c^	55.6
Friendster	2258	950	141	Failed^d^	N/A^c^	95.4

^a^ CPU details are described in Table 9

^b^ The GPU used for the experiments is a NVIDIA Volta V100

^c^ N/A indicates that the mentioned study didn’t report this dataset evaluation

^d^ The experiment hit the GPU memory limit

**Fig. 9:**
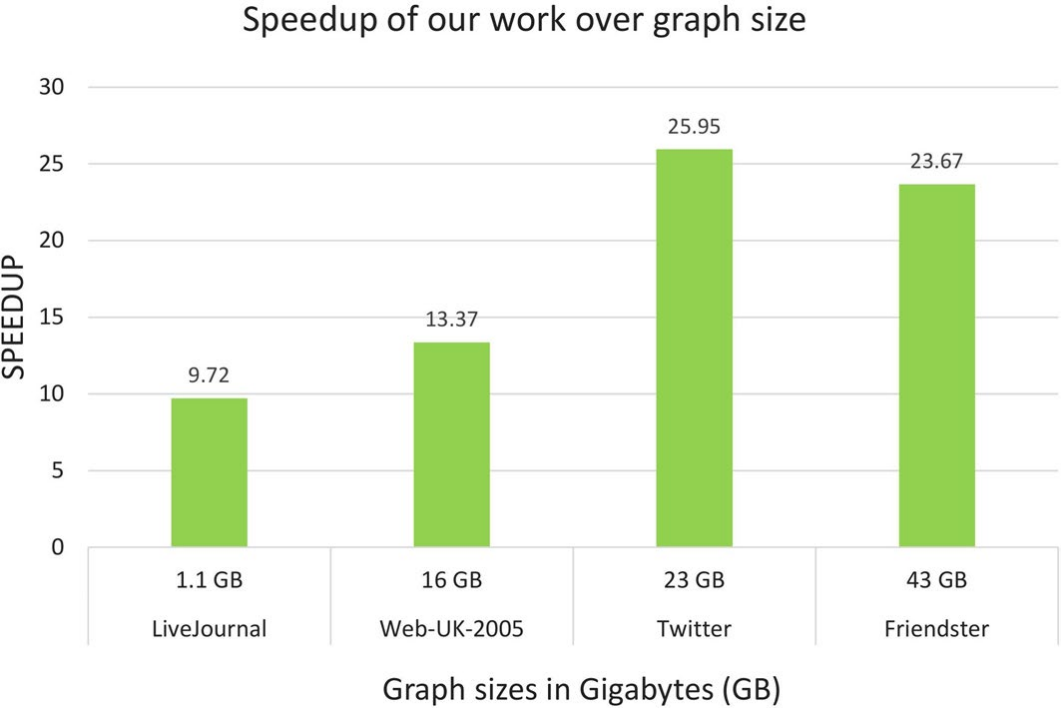
Speedup evaluation of the proposed implementation over the size of the graphs versus sequential execution. GB=Gigabytes

We also compared our algorithm with the GridGraph library [32], which uses a grid partition schema similar to ours and uses OpenMP for parallel execution. Our implementation demonstrates a performance improvement over the GridGraph library, with a potential speedup of 4x (Web-UK-2005). In addition to outperforming the CPU solutions, our FPGA implementation demonstrates significant performance improvements over the cuGraph library running on the selected GPU. The speedup achieved compared to the GPU reaches up to 4.5x with the Web-UK-2005 graph dataset. This dataset does not fit in the on-chip memory of the GPU and requires the use of the Unified Memory [39]. Data needs to be transferred back and forth between the host memory and the GPU on-chip memory, which incurs additional overhead and degrades performance. Moreover, the GPU experiments with bigger datasets (Twitter and Friendster) failed due to insufficient memory on the GPU board.

This shows the benefits of using an FPGA for graph processing tasks, especially when dealing with large datasets. We also compared our custom FPGA implementation with the PageRank algorithm available in the Vitis Library, as available in the literature [63], As shown in Fig. 8, our implementation achieves a speedup of about 28x over that library.

Our implementation demonstrates significant performance improvements over traditional CPU-based solutions, such as sequential execution, OpenMP parallel execution, and the GridGraph library, as well as over GPU-based solutions like the cuGraph library and pre-built FPGA libraries like Vitis’ library. The speedup achieved with our implementation highlights the benefits of using a custom FPGA implementation for graph processing tasks, especially when dealing with large datasets that do not fit in on-chip memory. These results demonstrate that our FPGA implementation is a suitable solution for accelerating graph processing tasks.

Figure 9 shows the speedup achieved by our proposed solution in relation to the size of the graph. Small graphs like LiveJournal show a speedup of about  10x. Bigger graphs like Web-UK-2005 show a speedup of about  14x, and larger graphs like Twitter show a speedup of about  26x. It is worth noting that speedup almost doubles from Web-UK-2005 to Twitter, even if Twitter’s size is about 1.5 larger than Web-UK-2005 (see Table 5). However, the performance boost that is achieved by the proposed work decreases when using the larger Friendster dataset. This saturation in performance has prompted us to investigate whether a multi-FPGA solution in a distributed system, like Hadoop, could potentially enhance the usage of FPGAs for large-scale graph processing.

**Fig. 10:**
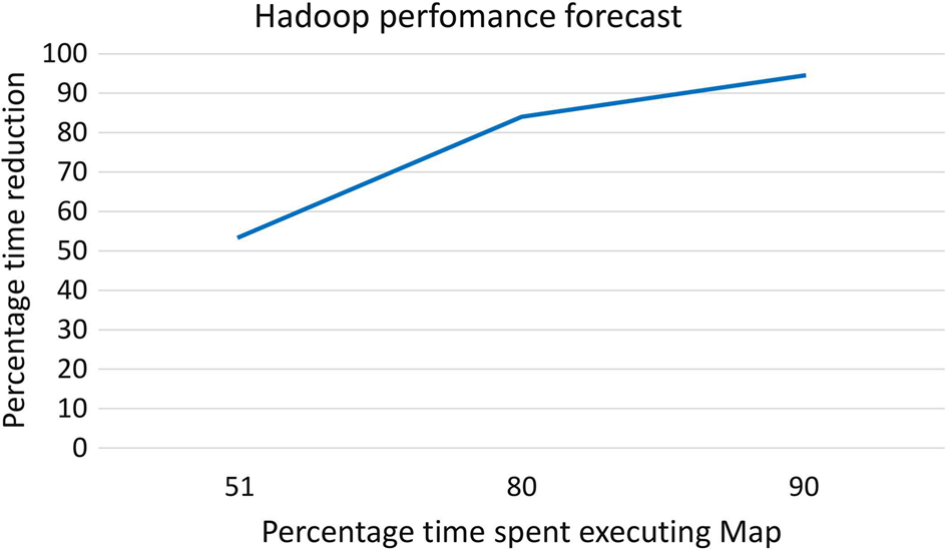
Time reduction achieved using FPGAs to accelerate graph processing compared to using a Hadoop cluster with no FPGA

Graphs bigger than Friendster exceed the computing capabilities of present-era machines. Hence, a possible solution to manage such large graphs is to divide them across multiple machines by means of distributed computing. Figure 10 presents the forecasts for integrating FPGA acceleration in a Hadoop-distributed cluster for large-scale graph processing. Profiling the code and using the results to evaluate Eq. (1) shows that using FPGAs is only effective if a significant amount of time is spent in the mapping phase. In the forecasts, we assume that most of the time is spent in the mapping phase, specifically 51% of the total time. Under these conditions, using the results from Table 10, assuming a worst-case scenario where FPGA accelerated nodes achieve only a 20x speedup compared to a CPU only nodes, and substituting them in Eq. (1), the forecasts show that it is possible to improve performance by reducing the total time by 54%. However, in a most realistic case scenario, most of the time is spent in the mapping phase, assuming 80% as a realistic assumption when using a partition method like GridGraph to minimise the data transfer. In this case, the time reduction achieved by a hybrid CPU-FPGA Hadoop cluster can grow to 84% compared to a CPU-only cluster. Figure 10 summarise these forecasts and also shows the best-case (but unlikely) scenario where 90% of the total execution time is spent in the mapping phase, showing that it is possible to achieve over 90% time reduction under best conditions.

## Conclusions

Large graph processing is an emerging field that deals with the analysis and manipulation of large and complex graphs, such as social networks, web graphs, road networks, and biological networks. With the increasing amount of data generated by these applications, the size of graphs is rapidly growing. As a result, the need for efficient and scalable graph processing techniques is becoming increasingly important. Combining the use of FPGAs with Hadoop for handling large graph datasets is a growing area of interest. FPGAs are specialised integrated circuits that can be customised for specific tasks and are highly efficient in executing graph processing algorithms. Hadoop is an open-source framework commonly used for distributed processing of large datasets. We show that utilising FPGA architecture in conjunction with graph partitioning can lead to high performance without limitations on the graph sizes, even when dealing with millions of vertices and billion of edges. In the case of the PageRank algorithm, for example, our optimised FPGA version is faster than any state-of-the-art implementation. The evaluation results demonstrate that our implementation outperforms GridGraph [32] by 2×, Cugraph [25] by 4.4× and VITIS LIB [4] by 26×. This highlights the efficiency of using custom FPGA implementations for graph processing tasks, particularly when dealing with large datasets that exceed the capacity of on-chip memory. Given that large-scale graphs can exceed the computational capabilities of a single machine, we analysed the benefits of our architecture in the context of distributed computing. The combination of FPGAs and distributed frameworks, such as Hadoop, can significantly enhance performance, particularly for large-scale datasets. The forecasts show that using FPGAs in a Hadoop cluster can reduce the processing time from 59% in the worst-case scenario to over 93% under the best conditions. Our current focus is on implementing additional optimisations to achieve even higher performance on large-scale graph processing.

## Data Availability

The datasets used and analysed during the current study are available from the corresponding authors upon reasonable request.

## Footnotes

1 We use the term “edge” to refer to arcs directed in either direction, although in graph theory the rigorous term for a directed edge is “arc”.
